# Extensive Emphysematous Necrotising Pancreatitis Presenting With Cullen’s Sign Despite a Normal Serum Amylase Level

**DOI:** 10.7759/cureus.109225

**Published:** 2026-05-19

**Authors:** Wael Abdelgawwad, Mohamed Eslam, Rawan Honeini, Noor Ali, Razan Honeini

**Affiliations:** 1 Emergency Medicine, United Lincolnshire Teaching Hospitals NHS Trust, Boston, GBR

**Keywords:** atypical presentation, cullen's sign, emphysematous pancreatitis, pancreatic necrosis imaging, severe pancreatitis

## Abstract

Emphysematous necrotising pancreatitis is a rare and life-threatening form of acute pancreatitis characterised by infected pancreatic necrosis with gas formation and a high risk of systemic complications. We report the case of a man presenting with a seven-day history of constipation, progressive abdominal distension, vomiting, and systemic deterioration, with periumbilical discoloration consistent with Cullen’s sign. Despite a normal serum amylase level, contrast-enhanced CT demonstrated extensive necrosis involving the neck and mid-body of the pancreas, gas locules within the peripancreatic region, and an acute necrotic collection in the lesser sac, consistent with emphysematous necrotising pancreatitis. His condition progressed despite supportive treatment, with interval imaging showing enlarging loculated intra-abdominal collections, mesenteric oedema, and vascular narrowing, and his course was further complicated by respiratory failure requiring intubation and probable aspiration pneumonia. This case highlights that severe necrotising pancreatitis may present with atypical biochemical findings and emphasises the importance of clinical suspicion, early cross-sectional imaging, serial reassessment, and multidisciplinary intensive care management.

## Introduction

Acute pancreatitis encompasses a spectrum of disease severity ranging from mild, self-limiting inflammation to severe disease associated with multi-organ failure and death [1]. Necrotising pancreatitis develops in a minority of patients but carries substantially greater morbidity and mortality, particularly when infected necrosis occurs [2,3]. A particularly severe form is emphysematous pancreatitis, in which gas is identified within necrotic pancreatic tissue, usually caused by gas-forming organisms and associated with an adverse prognosis [4]. Severe pancreatitis may be complicated by peripancreatic fluid collections, vascular complications, gastrointestinal oedema, and respiratory failure [5,6]. We present a case of extensive emphysematous necrotising pancreatitis presenting with Cullen’s sign despite normal serum amylase, highlighting the potential for atypical biochemical presentation in a rapidly progressive and life-threatening disease process.

## Case presentation

A 74-year-old man presented with a seven-day history of constipation, progressive abdominal distension, vomiting, and systemic deterioration. He reported a persistent sensation of incomplete defecation, with only minimal bowel motions despite taking senna for five days. He also described reduced appetite, poor oral intake, three episodes of vomiting, dark foul-smelling urine, and a sensation of incomplete bladder emptying.

On initial assessment, he was alert and oriented but appeared breathless. His temperature was mildly elevated, and oxygen saturation was 93% on room air. Abdominal examination demonstrated marked distension, absent bowel sounds, mild diffuse tenderness, and periumbilical discoloration later identified as Cullen’s sign (Figure 1).

**Figure 1:**
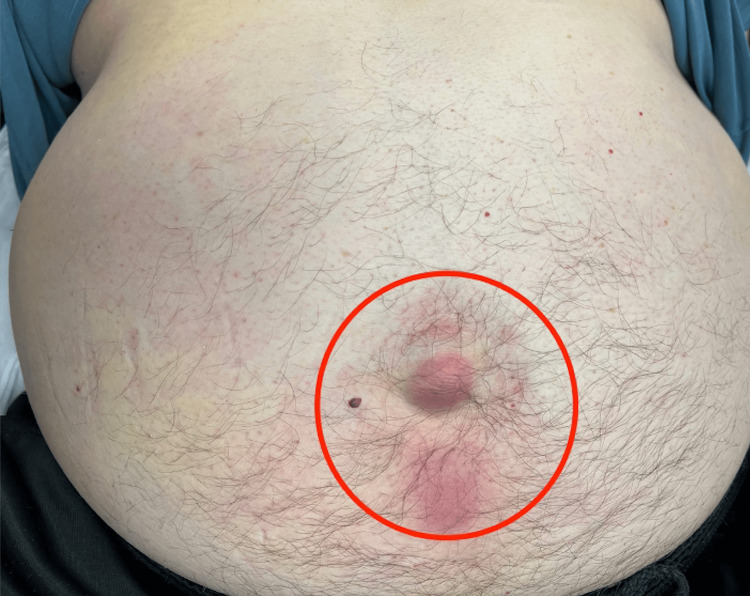
Cullen’s sign showing periumbilical bruising (red circle).

Initial laboratory investigations demonstrated normal serum amylase despite clinical concern for significant intra-abdominal pathology.

Given his concerning presentation, contrast-enhanced CT of the abdomen was performed. This demonstrated a complex gas-fluid collection measuring 7.2 × 5.2 cm in the lesser sac, free gas locules in the peripancreatic region, and necrosis involving the neck and mid-body of the pancreas (Figure 2). The remaining pancreatic parenchyma appeared oedematous. The collection was not encapsulated, consistent with an acute necrotic collection. The stomach was markedly fluid-filled, and nasogastric decompression was advised because of aspiration risk. Atelectatic change was also noted at the right lung base. Overall, the findings were in keeping with extensive necrotising pancreatitis with emphysematous changes and an acute necrotic collection in the lesser sac.

**Figure 2:**
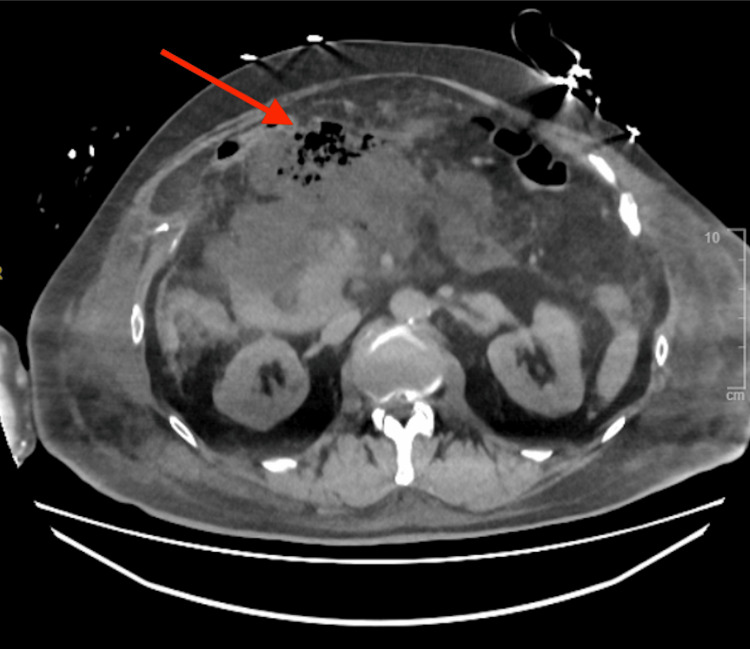
Axial contrast-enhanced CT scan showing necrosis within the neck and mid-body of the pancreas (red arrow).

The patient was admitted to the intensive care unit for close monitoring and organ support. He was managed with supportive care, intravenous antibiotics (ciprofloxacin and metronidazole), fluid management, and enteral nutritional support. Oesophagogastroduodenoscopy was performed to facilitate nasojejunal tube placement, which was successfully inserted and radiologically confirmed without procedural complication.

During admission, interval imaging demonstrated disease progression, with enlarging loculated intra-abdominal collections, increasing mesenteric oedema, and vascular narrowing. His clinical course was further complicated by respiratory deterioration requiring intubation and mechanical ventilation. Subsequent thoracic imaging demonstrated bilateral lower lobe consolidations and pleural effusions, in keeping with aspiration pneumonia.

Blood tests showed significantly elevated CRP (308 mg/L) and WBC count (17.6 × 10⁹/L) alongside a normal amylase level (76 U/L), as shown in Table 1. Although a normal amylase level can make acute pancreatitis less likely, it does not exclude severe or necrotising pancreatitis, particularly in the appropriate clinical and radiological context. These findings were also consistent with a severe inflammatory or surgical process. Concurrently, serial serum lactate levels were monitored over the course of one week, revealing a progressive rise from 2.0 mmol/L to 3.1 mmol/L, peaking at 5.7 mmol/L, before subsequently declining to 1.2 mmol/L. Blood cultures showed no growth within the first 24 hours and remained negative for both aerobic and anaerobic organisms at the end of the five-day monitoring period.

**Table 1:** Laboratory parameters. Hb: Haemoglobin; Cr: Creatinine; Na: Sodium; K: Potassium; GFR: Glomerular filtration rate; ALT: Alanine transaminase.

Laboratory Parameter	Value	Normal Range
WBC	17.6	4.5-11 × 10⁹/L
Platelets	344	150-400 × 10³/µL
Urea	21	2.1-8.5 mmol/L
Cr	162	59-104 µmol/L
Na	130	136-145 mmol/L
Hb	13.2	14-18 g/dL
K	3.6	3.6-5.2 mmol/L
Amylase	76	28-100 U/L
CRP	308	<5 mg/L
GFR	36	90-100 mL/min
Bilirubin	35	0-21 µmol/L
ALT	19	0-33 U/L
Alkaline phosphatase	116	30-130 U/L
Lactate	2	0.5-2.2 mmol/L

## Discussion

This case illustrates an aggressive and rapidly progressive form of necrotising pancreatitis with several clinically important features: Cullen’s sign on examination, gas within necrotic pancreatic tissue on imaging, and a normal serum amylase level despite extensive disease. His Modified Glasgow Score (Imrie Score) was 4/8. This signifies an increased risk for the development of systemic complications or pancreatic necrosis, warranting close monitoring, often in a high-dependency or intensive care setting. Together, these findings underscore the fact that life-threatening pancreatitis may occasionally present with atypical biochemical results and that diagnosis should not rely on serum amylase alone when the clinical picture is concerning.

Cullen’s sign is a classic but uncommon physical finding characterised by periumbilical ecchymosis resulting from the tracking of haemorrhagic fluid along fascial planes. Although it is not specific to pancreatitis, its presence should immediately raise concern for severe intra-abdominal pathology, including haemorrhagic or necrotising pancreatitis [7]. In this patient, the identification of Cullen’s sign was an important bedside clue to the severity of the underlying process.

An additional teaching point is the presence of normal serum amylase despite extensive pancreatic necrosis. Although elevated pancreatic enzymes are commonly used to support the diagnosis of acute pancreatitis, normal enzyme levels do not exclude the condition, particularly when presentation is delayed or when there is extensive pancreatic destruction. In such settings, imaging becomes essential, especially when clinical deterioration, abdominal signs, or systemic features are out of proportion to the biochemical findings.

The presence of gas within the necrotic pancreatic bed strongly suggested infected necrosis, consistent with emphysematous pancreatitis, a rare but highly morbid entity [1,2]. The patient’s subsequent progression with enlarging intra-abdominal collections, respiratory failure, and probable aspiration pneumonia reflects the aggressive systemic burden of severe necrotising pancreatitis. Walled-off necrosis typically develops around four weeks from the onset of disease and represents a more organised encapsulated stage [6]. The identification of gas within necrotic pancreatic tissue is highly indicative of infected necrosis, often resulting from colonisation by gas-forming organisms such as *Escherichia coli* or *Klebsiella* species, and is linked with poorer clinical outcomes and an increased risk of death [8,9].

This case therefore highlights the importance of early escalation of care, serial imaging in deteriorating patients, multidisciplinary coordination, nutritional support, and close monitoring for local and systemic complications.

## Conclusions

Emphysematous necrotising pancreatitis is a rare and life-threatening form of acute pancreatitis that may present with deceptively atypical biochemical findings. This case demonstrates that a normal serum amylase level does not exclude severe pancreatic necrosis, particularly in a patient with Cullen’s sign, systemic deterioration, and concerning abdominal findings. Early imaging, close interval reassessment, intensive multidisciplinary management, and prompt recognition of complications are critical to improving outcomes in this high-risk condition.
